# The Effects of Self-Monitoring Using a Smartwatch and Smartphone App on Stress Awareness, Self-Efficacy, and Well-Being–Related Outcomes in Police Officers: Longitudinal Mixed Design Study

**DOI:** 10.2196/60708

**Published:** 2025-01-28

**Authors:** Herman Jaap de Vries, Roos Delahaij, Marianne van Zwieten, Helen Verhoef, Wim Kamphuis

**Affiliations:** 1 Department of Learning and Workforce Development, The Netherlands Organisation for Applied Scientific Research, Soesterberg, Netherlands; 2 Department of Work Health Technology, The Netherlands Organisation for Applied Scientific Research, Leiden, Netherlands; 3 Department of Sustainable Productivity and Employability, The Netherlands Organisation for Applied Scientific Research, Leiden, Netherlands

**Keywords:** wearable electronic devices, ecological momentary assessment, psychological stress, psychological well-being, awareness, self-efficacy, occupational medicine, emergency responders, well-being, psychological, efficacy, stress, wearables, wearable device, smartwatch, smartphone app, app, sensor, sensor technology, police officers, questionnaire, stress awareness, stress management

## Abstract

**Background:**

Wearable sensor technologies, often referred to as “wearables,” have seen a rapid rise in consumer interest in recent years. Initially often seen as “activity trackers,” wearables have gradually expanded to also estimate sleep, stress, and physiological recovery. In occupational settings, there is a growing interest in applying this technology to promote health and well-being, especially in professions with highly demanding working conditions such as first responders. However, it is not clear to what extent self-monitoring with wearables can positively influence stress- and well-being–related outcomes in real-life conditions and how wearable-based interventions should be designed for high-risk professionals.

**Objective:**

The aim of this study was to investigate (1) whether offering a 5-week wearable-based intervention improves stress- and well-being–related outcomes in police officers and (2) whether extending a basic “off-the-shelf” wearable-based intervention with ecological momentary assessment (EMA) questionnaires, weekly personalized feedback reports, and peer support groups improves its effectiveness.

**Methods:**

A total of 95 police officers from 5 offices participated in the study. The data of 79 participants were included for analysis. During the first 5 weeks, participants used no self-monitoring technology (control period). During the following 5 weeks (intervention period), 41 participants used a Garmin Forerunner 255 smartwatch with a custom-built app (comparable to that of the consumer-available wearable), whereas the other 38 participants used the same system, but complemented by daily EMA questionnaires, weekly personalized feedback reports, and access to peer support groups. At baseline (T0) and after the control (T1) and intervention (T2) periods, questionnaires were administered to measure 15 outcomes relating to stress awareness, stress management self-efficacy, and outcomes related to stress and general well-being. Linear mixed models that accounted for repeated measures within subjects, the control and intervention periods, and between-group differences were used to address both research questions.

**Results:**

The results of the first analysis showed that the intervention had a small (absolute Hedges *g*=0.25‐0.46) but consistent effect on 8 of 15 of the stress- and well-being–related outcomes in comparison to the control group. The second analysis provided mixed results; the extended intervention was more effective than the basic intervention at improving recovery after work but less effective at improving self-efficacy in behavior change and sleep issues, and similarly effective in the remaining 12 outcomes.

**Conclusions:**

Offering a 5-week wearable-based intervention to police officers can positively contribute to optimizing their stress-related, self-efficacy, and well-being–related outcomes. Complementing the basic “off-the-shelf” wearable-based intervention with additional EMA questionnaires, weekly personalized feedback reports, and peer support groups did not appear to improve the effectiveness of the intervention. Future work is needed to investigate how different aspects of these interventions can be tailored to specific characteristics and needs of employees to optimize these effects.

## Introduction

### Background

First responders, such as police officers and firefighters, are regularly faced with highly demanding working conditions [1] such as threatening or emotionally demanding situations. These roles frequently involve irregular shifts and peak workloads, sometimes compounded by understaffing [2]. Such conditions can lead to increased stress levels [3], reduced mental well-being [1,4], sleep disturbances [5], and difficulties in maintaining a healthy lifestyle [6], thereby escalating the risks of long-term health issues [7] and absenteeism [8]. Although some of the inherent challenges of these professions are unavoidable, it could be beneficial for employees to gain insights into their own stress levels, recovery patterns, and lifestyle-related factors like sleep and physical activity [9]. Such personal monitoring could help them to improve their stress- and well-being–related awareness and self-efficacy to make behavioral adjustments in order to better manage demanding aspects of their professions and thus optimize their well-being.

The emergence of wearable sensor technology facilitates promising opportunities to monitor a broad set of health-related outcomes [10]. Besides the initial use cases of wearables in estimating daily physical activity levels [11] and sleep duration [12], the introduction of photoplethysmography sensors enabled the measurement of other metrics such as heart rate [13], heart rate variability (HRV) [14], oxygen saturation [15], and potentially blood pressure [16]. Wearable-based measurements such as sleep and resting HRV have already been linked to stress- and well-being–related outcomes in first responders [17,18], but are sometimes also used to estimate other relevant outcomes. Using measures such as HRV as inputs, wearable companies now also estimate mental stress levels [19,20] and physiological recovery [21] in an attempt to provide more easily interpretable feedback. Although conceptually useful, in consumer-available wearables this is almost exclusively done via black box algorithms and the created measures do not always correlate optimally with similar subjective outcomes [22]. Despite these limitations and need for more scientific evaluation of the performance of these metrics, the wearables that utilize them are already broadly commercially available and adopted and are being integrated into wearable-based interventions [23]. In these wearable-based interventions, individuals use real-time data from physiological measurements to improve their health and performance, also known as biofeedback [24]. Given the continuing adoption of these wearables, it is important to understand the impact of these devices on behavior change and stress- and well-being–related outcomes.

### The Effectiveness of Wearable-Based Interventions

Wearable-based interventions can be supportive at increasing physical activity [25], reducing body weight [25-27], and improving sleep outcomes [28]. Regarding the application of wearables to reduce stress and support mental well-being, most research efforts are focused on the modeling of stress-related outcomes using wearables [29]. Although studies show that wearable-based stress detection is feasible, this is especially true in controlled (eg, laboratory) settings, whereas challenges remain for accurate measurement in real-life circumstances [30]. A few studies have been performed investigating the effectiveness of wearable-based interventions to reduce stress and improve well-being, and they found some promising results [31-33]. A recent review that surveyed the broader literature on this topic showed that these effect studies tend to assess acute stress responses in laboratory settings, often in young (student) populations [34]. The authors concluded that there is therefore a need for more studies that investigate the effects of longitudinal wearable-based interventions on stress- and well-being–related outcomes in a natural, real-life context.

To improve our understanding of the effects of wearable-based interventions, it is not only helpful to gain insight into their direct impact on outcomes such as stress and mental well-being, but also in the possible precursors to those outcomes. Effects on these precursors could be meaningful even in the absence of short-term effects on the primary outcomes and may substantiate why the found effects occur. With regard to these precursors, the Health Belief Model proposes that health-related awareness (eg, on the susceptibility for and severity of the potential health problem) and perceived self-efficacy to cope with the situation are necessary to come to action and change behavior, which in turn may improve health-related outcomes [35,36]. Metacognitive awareness (MCA) has been shown to be related to learning [37] in general and learning about coping with stress specifically [38]. MCA is defined as the ability to reflect upon, understand, and regulate one’s learning [37]. This concept distinguishes between insight in one’s responses and potential causes and ways to regulate these responses. MCA helps people to develop strategies to change their behavior in accordance with the situation.

Self-efficacy is considered a key mechanism through which behavioral change can be observed [39]. This has also been shown for the way people cope with stress [40]. People’s beliefs about their ability to change behavior is determined, among other factors, by mastery experiences (succeeding at changing behavior) and vicarious learning (seeing other succeed). Daily feedback through monitoring can provide valuable insight into how to change behavior and mastery of these behaviors in attainable steps.

Since most wearables are designed to improve their users’ awareness of their own health-related behavior and provide them with leads on what needs to change and how they are progressing, improvement in health outcomes may be preceded by increased awareness and self-efficacy. Some studies have confirmed that wearable-based interventions can indeed positively influence health-related awareness [41] and self-efficacy in patient populations [42,43], but it is unknown to what extent these findings translate to occupational settings. Health-related behavior change interventions generally require a duration of months to years in order to ingrain the new behaviors [44], but when studying awareness and other precursors of behavior change, a shorter duration (eg, weeks) is sufficient.

Therefore, the first research question that this study addresses is the following:

Research question 1: Does offering a 5-week wearable-based intervention improve awareness, self-efficacy, and stress- and well-being–related outcomes in police officers in comparison to a control period?

### The Potential Added Value of Enhanced Feedback and Implementation

For policymakers considering the deployment of wearable-based interventions, it is also helpful to know to what extent it is sufficient to use an “off-the-shelf” solution, where consumer-available wearables are directly deployed to the target group, or whether it is necessary, for instance, to provide (1) more extensive feedback and (2) (peer) support for the intervention to be effective. Based on self-efficacy theory, the latter could be of added value as it provides potential for vicarious learning and supports awareness of ways to attain mastery experience, whereas the former may contribute to the development of increased awareness.

First, it is possible to additionally collect subjective data via brief daily questionnaires, also known as ecological momentary assessment (EMA), and utilize the data to provide feedback on outcomes that cannot be measured using wearables. These self-report data can also be used to provide contextual and subjective information to the objective wearable-based measures [45]. Providing this more enriched feedback could potentially have beneficial effects on awareness as it links personal experience to objective measurement and provides information on the behavioral context that affects stress and well-being. This in turn can inform users on strategies to attain behavior change.

Second, organizing peer support groups can be a way to facilitate collaborative reflection and group learning [46]. Literature on the effectiveness of peer consultation groups in a preventive occupational health context is scarce, but there are indications that these can positively contribute to recovery from mental health conditions [47-49]. Although different from peer consultation through the guidance of more experienced professionals, peer supervision has also been shown to have positive effects on self-awareness and self-efficacy in occupational settings [50,51]. It is therefore possible that utilizing peer support groups may benefit the effectiveness of wearable-based interventions that aim to achieve health-related behavior change and optimization of stress- and well-being–related outcomes.

To contrast the effects of a condition that is enriched by both enhanced feedback and added peer support to a more simple “off-the-shelf” intervention that just utilizes a consumer-available wearable, the second research question is as follows:

Research question 2: Is offering a 5-week wearable-based intervention that is extended by EMA, personalized weekly feedback, and peer support meetings (“Wearable+”) more effective at improving awareness, self-efficacy, and stress- and well-being–related outcomes in police officers than an “off-the-shelf” (“Wearable”) intervention?

## Methods

### Ethical Considerations

The study protocol was approved (case 2022‐100) by the internal Research Ethics Committee of the Netherlands Organisation for Applied Scientific Research (TC-nWMO). Participation occurred voluntarily based on informed consent, and participants could opt out at any time without further consequences. Data were collected in a custom-built app that allowed for full control over the collected data without using consumer cloud services. During data collection, data were pseudonymously stored by using participant identifiers and anonymized after the data collection was completed by deleting the participant identifiers. Participants did not receive monetary compensation, but in each participating police team a lottery was held among the participants that adhered to the measurement protocol, with one wearable awarded per team.

### Design

**Figure 1.**
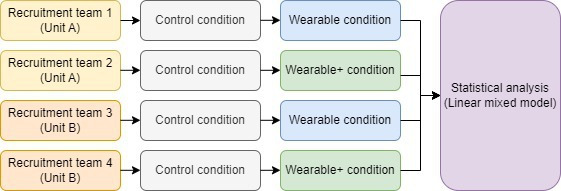
A mixed design was applied in this study, combining elements of within- and between-subject designs.

A mixed research design was applied, combining elements of a within-subject design with those of a between-subject design (Figure 1). In this study, 2 teams from 2 separate units of the Dutch police force were recruited. Of the teams in each unit, one team enrolled into the Wearable condition, whereas the other team enrolled into the Wearable+ condition (the between-subject comparison). Before undergoing the respective 5-week interventions, the participants in each team would first partake in a 5-week baseline control period (the within-subject comparison). This approach ensured that we would have a well-comparable control condition. No a priori stratification methods were applied, but *χ*^*2*^ tests to assess possible between-group differences in gender, age group, and wearable ownership before their participation were performed afterwards.

### Participants

The recruitment of participants was initiated in 2 teams from 2 units of the Dutch police. In each unit, police officers of one team were recruited for the Wearable condition, whereas the police officers of the other team were recruited for the Wearable+ condition. In each team, up to 25 participants were recruited (up to 100 participants in total). This clustered recruitment method ensured geographical distribution of participants while preventing contamination of the different interventions within teams. Due to a lack of response in one team (the Wearable condition team in the rural unit), participants were also recruited from an additional team within that same unit.

In each team, recruitment was done via a combination of posters, flyers, a mention of the study during a team meeting, and an email to all police officers. Interested police officers would use an online tool to check if they met the inclusion criteria, which meant that they were (1) aged 18 years or older, (2) executive police officers, and (3) willing to put aside their own wearable during the duration of the study if they owned one. The police officers then received an information brochure about the study and filled in an online informed consent form before they received their baseline questionnaire and started their participation in the 5-week control period. To stimulate participant adherence during the intervention condition, within each team, a lottery was held that included the participants that collected complete daily data for at least 80% of their participation period and filled in all 5-weekly outcome questionnaires, where the winning participant got to keep the wearable that was used during the study.

### Intervention

#### Control Condition

After filling in their baseline questionnaire, all participants first enrolled in a 5-week control period. Participants were instructed to live their lives as usual but to not use their own wearable (eg, smartwatch) if they owned one.

#### Wearable Condition

**Figure 2.**
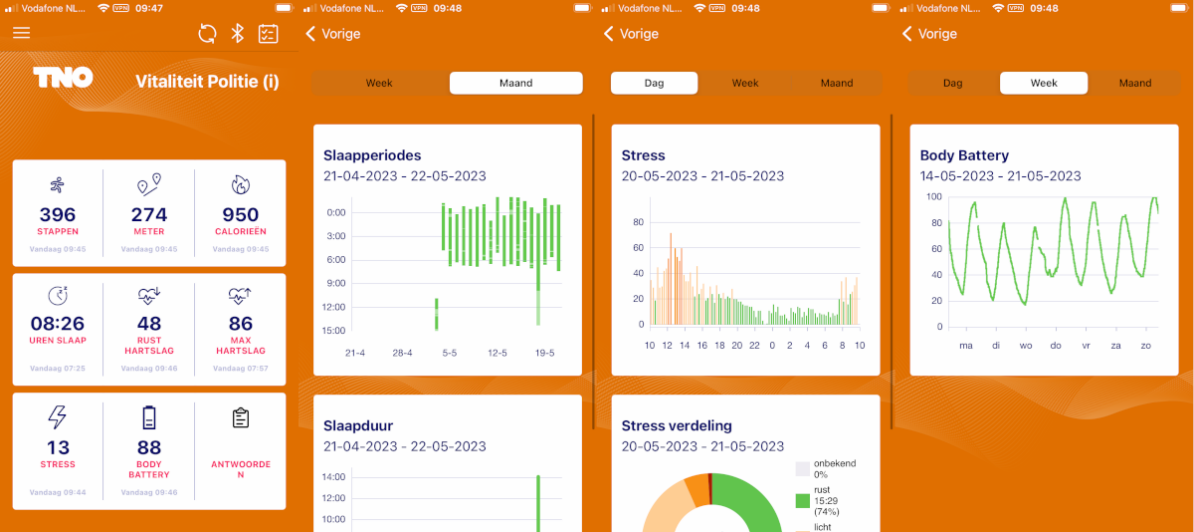
Screenshots of the (Dutch-language) Sensorium smartphone app that used the Garmin Health software development kit to directly retrieve data from the Garmin Forerunner 255. From left-to-right, the screenshots display the home screen dashboard (the version of the Wearable+ condition, which includes answers to the ecological momentary assessment questionnaires on the bottom right corner), a monthly view of sleep periods, a daily view of physiological stress, and a weekly view of the “body battery” metric, which estimates the individual’s energy levels.

After the control period, the participants that enrolled into the Wearable condition received an “off-the-shelf” intervention that was based on just a consumer-available smartwatch. Due to the sensitive profession of the participants in combination with Dutch privacy laws, it was not possible to use a platform that stores data outside the European Union. To accommodate this need, a Garmin Forerunner 255 smartwatch was used in combination with a smartphone app that was approved for use with the Dutch police (built by Sensorium 69 BV), as it directed data directly from the device to self-controlled servers. The app displayed much of the same data as the Garmin Connect app that regular Garmin consumers use (Figure 2). The displayed wearable-based metrics were steps, walking distance, active calories, total sleeping time, resting and maximum heart rate, physiological stress, and a “body battery” metric that estimates the individual’s energy levels. The wearable also measured the daily HRV status and (after 3 weeks) provided feedback on the device itself regarding the extent in which the participant’s resting physiology was in a normal state; if it was not, the device would advise the individual to take it easy that day. Other functions such as sports tracking were also only available on the wearable device itself.

During the 5-week intervention period, Wearable condition participants used the wearable and app at their own discretion. The researchers were available via email and a messaging app to help with technical issues, but no other support was given.

#### Wearable+ Condition

The Wearable+ condition represented an enriched situation, where a consumer-available wearable was complemented by additional feedback and support. The composition of the Wearable+ condition was based on a prior (unpublished) study that was done with the Dutch police force, where police officers tested the “off-the-shelf” solution of the current Wearable condition in this study and described how the intervention could be improved to more optimally fit their needs and working context.

Participants enrolled in the 5-week Wearable+ condition used the same Garmin Forerunner 255 that the participants in the regular Wearable condition used. The accompanying custom-built smartphone app was also the same, but it included a feature that allowed the Wearable+ condition participants to also fill in a brief daily EMA questionnaire. The daily EMA questionnaire was available each day during the late afternoon, and participants were instructed to fill it in before they went to bed. The questionnaire contained items on the type of shift that they worked that day, as well as their perceived stress, need for recovery, affective state (valence and arousal), and an optional custom annotation to report incidents or issues that they thought were potentially relevant, where they could write a brief note of up to 35 characters.

The EMA questionnaire data were visible for the participants in their app and were also used for more comprehensive personalized feedback reports that the participants received on a weekly basis. In the clickable weekly feedback report that offered tabs with alternative views and metrics (based on an R Notebook), the data of the subjective EMA outcomes were combined with the objective wearable-based outcomes in order to provide enriched contextual information and compare trends. In addition, textual messages were added to support participants in reflecting on the data. The contextual information could help participants in the interpretation of the wearable-based outcomes, for instance, to better understand why certain scores were abnormally low or high and thus identify potentially actionable associations. When little EMA data was available, the participant was reminded to fill in their daily EMA questionnaire, as it would improve the richness of the feedback report. In the second-to-last week of the intervention period, the report also included a personalized reflection on the data (eg, highlighting relevant trends or associations) and, if possible, advice from the researchers (HJdV, HV, and MvZ) based on the data of the participant. This advice was seen as a proxy for a future algorithm-based function that provides overarching personalized advice based on the broader range of available data sources.

Finally, peer support groups were organized twice per week, where researchers (HV and MvZ) were also available, for instance, to help with the interpretation of the weekly reports. In the peer support groups, participants primarily discussed their personal experiences, for example, with the smartwatch, personal feedback reports, and potential lifestyle changes that they were considering or executing. The personal data of the feedback reports of the participants were not publicly shared by the researchers, but participant questions on the interpretation of certain outcomes were discussed in general terms in order to optimize participants’ understanding of the feedback reports. Additionally, participants could contact the researchers via email, telephone, or an online video call on an individual basis for specific questions about their own device or data.

### Data Collection

#### Overview

The intervention effects were assessed via an online questionnaire that was administered at baseline (T0), after the control period (T1), and after the intervention period (T2). For descriptive purposes, questions on the participants’ gender, age category (under 30 years old, 30-45 years old, older than 45 years), and whether they already owned a wearable before participating in the study were included. Wearable ownership was also used as a covariate during hypothesis testing to correct for potential differences in outcome variables among participants that did or did not own a wearable prior to their participation.

#### Metacognitive Awareness About Sleep, Stress, and Activity Assessment

To determine whether the intervention influenced the participants’ stress- and well-being–related awareness, we used the Metacognitive Awareness about Sleep, Stress, and Activity Assessment (MASSAA). This scale was developed in a pilot study in preparation for this study for the Dutch police [52]. Items are scored on a 7-point Likert scale ranging from “completely disagree” to “completely agree.” Items fall into 3 categories that are in line with the concept of MCA, namely awareness about responses, causes, and regulation. After performing post hoc factor analysis on the collected data, the responses to the used awareness and cause subscales were found to be very highly interrelated, after which these subscales were combined to minimize the number of performed analyses. Reliability of the subscales was good. Therefore, a total of 6 MASSAA subscales were assessed, namely stress awareness and cause (α=.87), stress regulation (α=.91), sleep awareness and cause (α=.86), sleep regulation (α=.93), physical activity awareness and cause (α=.90), and physical activity regulation (α=.92). The complete MASSAA questionnaire (both the original Dutch items and their English translations) can be found in Multimedia Appendix 1.

#### Self-Efficacy

Self-efficacy was measured via a series of propositions, which were scored on a 5-point Likert scale that ranges from “completely disagree” to “completely agree,” in order to assess if the intervention also affected the individual’s perceived capacity to execute behaviors necessary to produce specific outcomes [39] and therefore possibly indirectly affected stress-related outcomes [53]. Separate scales were used. First, a scale for behavior change relating to stress, recovery, sleep and activity was used. This scale was developed in a pilot study in preparation for this study. Reliability was adequate (α=.79). Furthermore, self-efficacy scales with respect to stress resilience (α=.82), coping (α=.83) and tasks (α=.86) were included [54]. For completeness, all used self-efficacy scales and items can be found in Multimedia Appendix 2.

#### Stress and Well-Being

Several stress- and well-being–related outcomes were also monitored. The participants’ perceived need for recovery during and after work were queried using a series of propositions that were scored on a 5-point Likert scale that ranges from “completely disagree” to “completely agree” [55]. Reliability was good, with an α of .79 and .87 for recovery during and after work, respectively. A 3-item subscale for sleep issues regarding waking up too early, sleeping through, and having trouble falling asleep was also included and scored on a 5-point Likert scale, ranging from “never” to “always.” Reliability was adequate (α=.74). The subscales for recovery during and after work, as well as for sleep issues, can be found in Multimedia Appendix 2. For the measurement of the emotional, psychological and social well-being of participants, the 14-item Dutch version of the Mental Health Continuum-Short Form (MHC-SF) was administered [56]. The MHC-SF asks how often the respondent experienced a range of well-being–related feelings during the last month and is scored on a 6-point Likert scale that ranges from “never” to “daily.” Reliability was good (α=.92). Finally, the 10-item version of the Perceived Stress Scale, 10-item version (PSS-10) was taken [57]. The PSS-10 inquires about the frequency of the occurrence of stress-related feelings and thoughts during the last month, and it is evaluated on a 5-point Likert scale that ranges from “never” to “very often.” Reliability was good (α=.78).

### Data Analysis

Data management and statistical analyses were performed in RStudio (version 2023.06.2) [58] using R (version 4.2.2; R Foundation for Statistical Computing) [59].

#### Data Management

The data of participants that used their own wearable during the study or that did not fill in all outcome questionnaires (eg, dropouts or nonresponse) were filtered out. For the description of the participants’ gender, age, and wearable ownership prior to the study, as well as the statistical testing for potential between-group baseline differences (via *χ*^*2*^ tests) and the development of supporting data visualizations, the observed data were used. For hypothesis testing, the data were first standardized at the grand mean [60].

#### Hypothesis Testing

Hypothesis testing was done using version 1.1‐33 of the lme4 R package, which allows for statistical testing with linear mixed models (LMMs) [61]. For each of the 15 awareness, self-efficacy, and well-being–related outcomes, modeling was performed using a 3-step process.

For analysis 1, a first model was formed based on solely the confounding variable wearable ownership prior to participating in the study as a fixed effect and the participant identifier as a random effect to account for repeated measures within-subject. Second, the model was extended by including the main variables of interest “time” (a binary label for the repeated measures before and after the control and intervention periods) and “period” (labels for the control and intervention periods). The third and final model was then extended by the interaction effect of time and period, which assesses if the changes over time differed between the control and intervention periods; it is therefore the key focus for answering research question 1. For each model, the marginal *R*^2^ (m*R*^2^) and conditional *R*^2^ (c*R*^2^) [62], as well as the difference (Δ) in each in comparison to the previous step, were described. For the final model, absolute values of Hedges *g* statistic were also calculated to estimate the observed effect size, along with its 95% CI. In the interpretation of Hedges *g*, effects below 0.20 were considered to be “very small,” between 0.20‐0.49 as “small,” between 0.50‐0.79 as “medium,” and above 0.8 as “large” [63]. To accommodate the interpretation of the results, a data visualization with interaction plots for each of the 15 outcomes was created.

For analysis 2, this same approach was used, but also included the main effects of the “group” variable (Wearable or Wearable+) in step 2 and the 3-way effect of time, period, and group in step 3. The latter evaluates if the effectiveness of the intervention in comparison to the control condition differed between the Wearable and Wearable+ groups and therefore contributes to answering research question 2.

## Results

### Overview

A total of 95 participants were recruited (48 Wearable, 47 Wearable+), of which 16 participants (7 Wearable, 9 Wearable+) were excluded from statistical analysis. Of the excluded participants, 12 (3 Wearable, 9 Wearable+) used their own private wearable during the control period, 2 (Wearable) dropped out, and 2 (Wearable) did not fill in the final (T2) questionnaire. The data of the remaining 79 participants—who were predominantly males (n=57, 72%) in the age category of 30-45 years (n=46, 58%) and did not own a wearable before participating in the study (n=47, 59%)—were analyzed. Descriptive statistics of the analyzed participants of both intervention groups and the total sample are described in Table 1. No statistically significant baseline differences between the Wearable and Wearable+ groups in gender, age category, and wearable ownership prior to participation were identified based on *χ*^*2*^ testing.

**Table 1.** Descriptive statistics of the analyzed participants in the Wearable group, Wearable+ group, and total sample.

		Wearable, n (%)	Wearable+, n (%)	Total, n (%)
Participants		41 (52)	38 (48)	79 (100)
Gender				
	Male	28 (68)	29 (76)	57 (72)
	Female	13 (32)	9 (24)	22 (28)
Age (years)				
	<30	12 (29)	4 (11)	16 (20)
	30‐45	21 (51)	25 (66)	46 (58)
	>45	8 (20)	9 (24)	17 (22)
Wearable owner				
	False	27 (66)	20 (53)	47 (59)
	True	14 (34)	18 (47)	32 (41)

### Analysis 1: Effect of the Intervention Conditions Against the Control Condition

**Figure 3.**
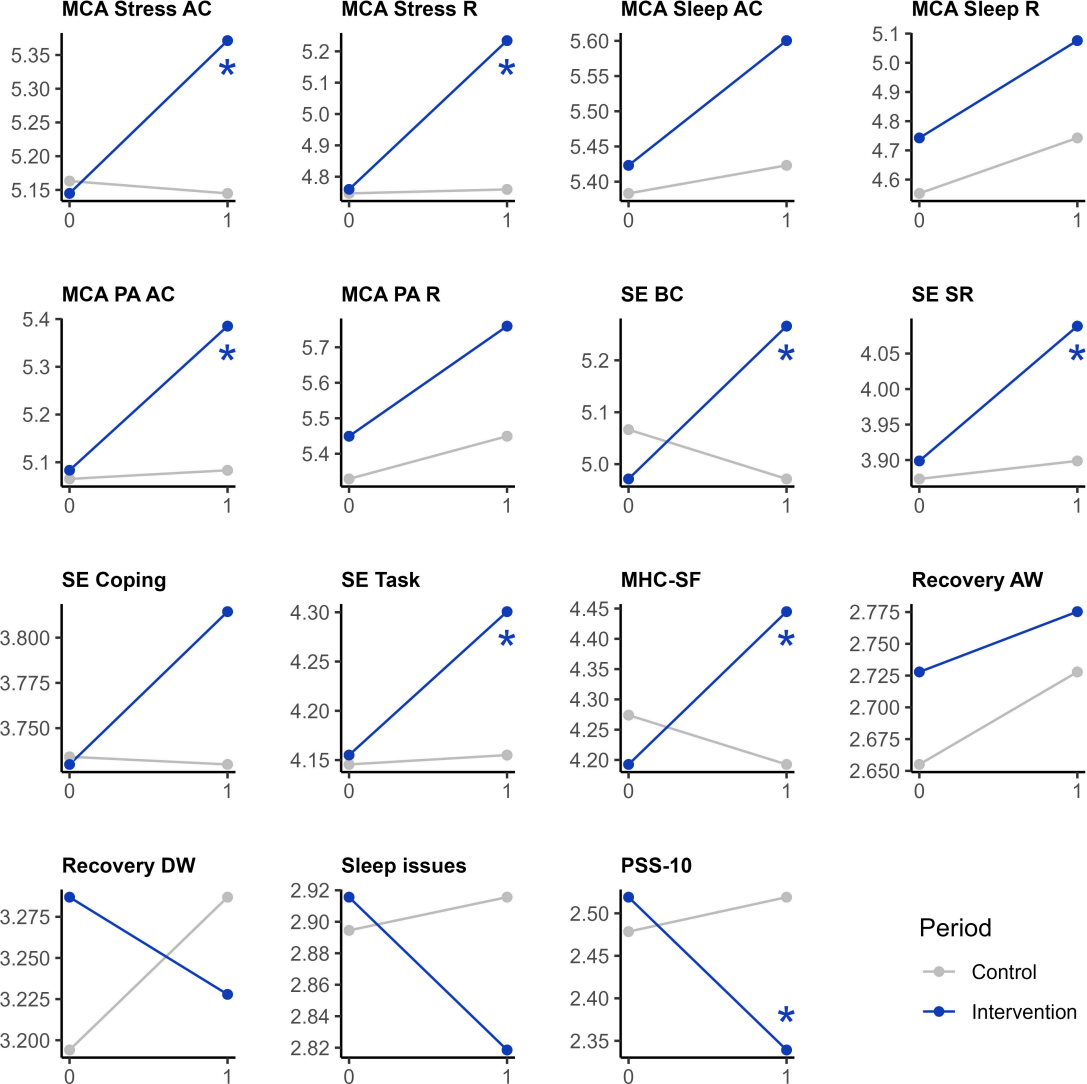
Interaction plots to support the interpretation of the results of analysis 1, which investigated the effectiveness of a wearable-based intervention in comparison to a control condition. For each of the 15 awareness-, self-efficacy–, and well-being–related outcomes, the mean scores before (“0” on x-axis) and after (“1” on x-axis) the control (gray) or intervention condition (blue) are visualized. Interaction plots with statistically significant (*P*<.05) 2-way interaction effects are annotated (*). AC: awareness and cause; AW: after work; BC: behavior change; DW: during work; MCA: metacognitive awareness; MHC-SF: Mental Health Continuum-Short Form; PA: physical activity; PSS-10: Perceived Stress Scale, 10-item version; R: regulation; SE: self-efficacy; SR: stress resilience.

LMMs were created for each of the 15 awareness-, self-efficacy–, and well-being–related outcomes. Statistically significant 2-way interaction effects were found in 8 outcomes, namely MCA stress awareness and cause (*P*=.04, m*R*^2^=.02, c*R*^2^=.59, Hedges *g*=0.30, 95% CI 0.01-0.58), MCA stress regulation (*P*=.002, m*R*^2^=.02, c*R*^2^=.66, Hedges *g*=0.41, 95% CI 0.16-0.66), MCA physical activity awareness and cause (*P*=.03, m*R*^2^=.07, c*R*^2^=.64, Hedges *g*=0.30, 95% CI 0.04-0.57), self-efficacy behavior change (*P*=.002, m*R*^2^=.06, c*R*^2^=.73, Hedges *g*=0.37, 95% CI 0.13-0.60), self-efficacy stress resilience (*P*=.04, m*R*^2^=.03, c*R*^2^=.71, Hedges *g*=0.25, 95% CI 0.01-0.50), and self-efficacy task (*P*=.03, m*R*^2^=.02, c*R*^2^=.68, Hedges *g*=0.29, 95% CI 0.04-0.54), as well as the MHC-SF (*P*<.001, m*R*^2^=.02, c*R*^2^=.74, Hedges *g*=0.40, 95% CI 0.17-0.63) and PSS-10 (*P*<.001, m*R*^2^=.02, c*R*^2^=.71, Hedges *g*=0.46, 95% CI 0.22-0.70). The detailed results for the 3-step LMM process for each of the 15 outcomes is available in Multimedia Appendix 3. The interaction plots in Figure 3 summarize these findings by visualizing the average scores before (“0” on x-axis) and after (“1” on x-axis) the control (gray) and intervention (blue) conditions, and labeling the statistically significant interaction effects.

For each of the 8 statistically significant outcomes, the wearable-based intervention had a favorable effect in comparison to the control condition. For 6 of the remaining 7 nonstatistically significant outcomes, the direction of the effect also favored the intervention, with the exception of recovery during work. Therefore, the observed effects consistently favored the wearable-based intervention over the control period, for which the statistically significant effects were estimated to be “small” (Hedges *g*=0.25-0.46) in size. In these outcomes, the fixed effects (the control or intervention period) explained between 2% and 7% of the variance, whereas the full model including the random effect (the participant identifier) explained between 59% and 74% of the variance in the outcomes.

### Analysis 2: Effects in the Wearable+ Group Compared to the Wearable Group

**Figure 4.**
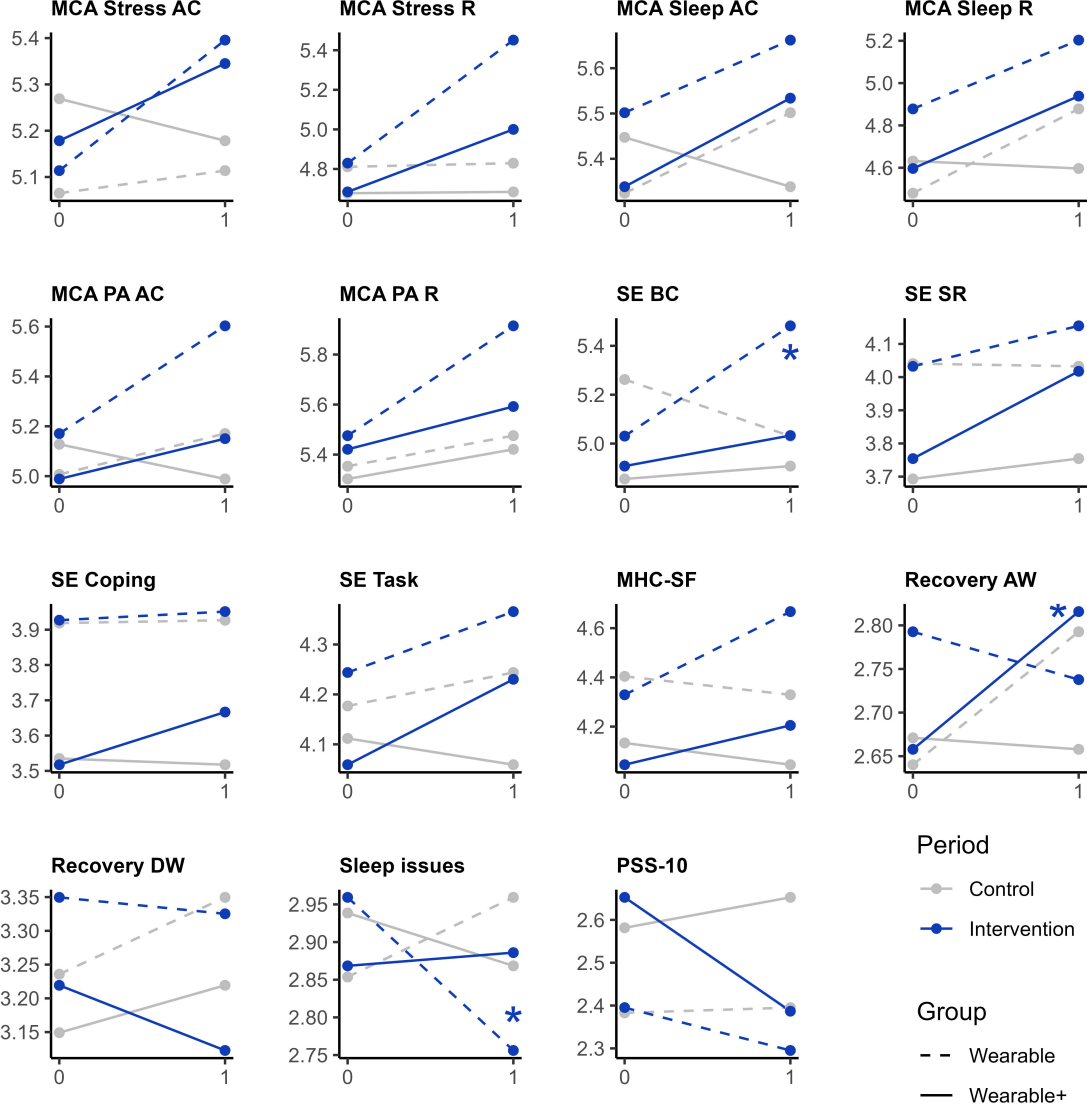
Interaction plots to support the interpretation of the results of analysis 2, which investigated possible differences in the effectiveness in 2 wearable-based interventions. For each of the 15 awareness-, self-efficacy–, and well-being–related outcomes, the mean scores before (“0” on x-axis) and after (“1” on x-axis) the control (gray) or intervention condition (blue) are visualized, for both the Wearable (striped line) and Wearable+ (solid line) groups. Interaction plots with statistically significant (*P*<.05) 3-way interaction effects are annotated (*). AC: awareness and cause; AW: after work; BC: behavior change; DW: during work; MCA: metacognitive awareness; MHC-SF: Mental Health Continuum-Short Form; PA: physical activity; PSS-10: Perceived Stress Scale, 10-item version; R: regulation; SE: self-efficacy; SR: stress resilience.

In the LMMs that were created for analysis 2, statistically significant 3-way interaction effects were found in 3 of 15 outcomes, namely self-efficacy behavior change (*P*=.02, m*R*^2^=.10, c*R*^2^=.75, Hedges *g*=0.58, 95% CI 0.11-1.05), recovery after work (*P*<.05, m*R*^2^=.01, c*R*^2^=.83, Hedges *g*=0.41, 95% CI 0.04-0.77), and sleep issues (*P*=.03, m*R*^2^=.02, c*R*^2^=.77, Hedges *g*=0.45, 95% CI 0.02-0.88). The detailed results for the 3-step LMM process are available in Multimedia Appendix 4. The interaction plots in Figure 4 summarize these findings by visualizing the average scores before (“0” on x-axis) and after (“1” on x-axis) the control (gray) and intervention (blue) conditions for both the Wearable (striped line) and Wearable+ (solid line) groups and labeling the statistically significant interaction effects.

Based on the interaction plots of the outcomes with statistically significant 3-way interaction effects (Figure 4), the enhanced Wearable+ intervention was more effective than the “off-the-shelf” Wearable intervention for improving recovery after work, but less effective for improving self-efficacy in behavior change and perceived sleep issues. However, the plots of these 3 outcomes all showed a noticeable change during the control period, all of which benefited the intervention group, which demonstrated statistically significant improvements in the respective analyses. Therefore, these changes during the control period may have influenced the results for these outcomes. Furthermore, the broad 95% CIs of the estimated effect sizes, compared to those in analysis 1, indicate decreased certainty in the magnitude of the reported effects. The results of analysis 2 should therefore be interpreted more cautiously than the results of analysis 1.

## Discussion

### Principal Results

#### The Effectiveness of Wearable-Based Interventions (Analysis 1)

The first aim of this study was to assess whether offering a 5-week wearable-based intervention would improve awareness, self-efficacy, and stress- and well-being–related outcomes in police officers in comparison to a control period. The results of analysis 1 showed that the wearable-based intervention consistently benefited the participants in 8 of the 15 outcomes, namely MCA of stress awareness and cause, MCA of stress regulation, MCA of physical activity awareness and cause, self-efficacy in behavior change, self-efficacy in stress resilience, self-efficacy in task execution, well-being, and stress. This means that the wearable-based intervention had a direct positive influence on the primary stress and well-being outcomes, as well as several aspects of MCA and self-efficacy, which were seen as precursors to relevant behavior change, based on the Health Behavior Model [35,36].

The effect sizes of the 8 statistically significant outcomes were estimated to be “small” (Hedges *g*=0.25-0.46). The variance that could be explained by the fixed intervention effects only was 2%‐7% (based on the m*R*^2^), whereas the variance that could be explained when also including the random effects of participant identifiers ranged from 59%‐74% (based on the c*R*^2^). This indicates that there are large interindividual differences among participants that can potentially play a role in how individuals respond to the intervention. Future studies with a larger sample size are needed to understand which individual factors influence the differential effectiveness of the intervention and the extent to which personalized approaches could enhance the effectiveness of the intervention, taking into account the variability among participants.

Based on the results of analysis 1, offering a 5-week wearable-based intervention has a small but positive impact on stress- and well-being–related outcomes in police officers. Further studies with a larger sample size are needed to explore if the intervention is particularly effective for certain subgroups and whether more personalized approaches could better accommodate interindividual differences, thereby potentially increasing its overall effectiveness.

#### The Potential Added Value of Enhanced Feedback and Implementation (Analysis 2)

This study also aimed to investigate if offering an enriched wearable-based intervention that is extended by additional EMA questionnaires, personalized weekly feedback, and peer support meetings would be more effective than offering a more basic “off-the-shelf” wearable-based intervention. Analysis 2 showed mixed results, where the enriched intervention was more effective than the basic intervention at improving recovery after work, less effective for self-efficacy in behavior change and sleep issues, and similarly effective for the other 12 outcomes.

Similar to the results of analysis 1, the estimated effect sizes of the differences between the Wearable and Wearable+ intervention groups were small (Hedges *g*=0.41-0.45) to medium (0.58), although the much larger confidence intervals in analysis 2 suggest that these results should be interpreted more cautiously. This, combined with the observation that changes in the control period might have exacerbated the between-group effects in the 3 statistically significant outcomes, warrants the conclusion that confirmation in future studies with a larger sample size is desired before policy decisions are made based on these insights. Due to the again observed large differences between the variance that were explained by the effects of the 2 interventions (1%‐10%) and by the full models (75%‐83%), exploration regarding whether certain individuals may benefit more from either intervention and whether additional personalization may increase their overall effectiveness would be interesting.

In view of these findings, the enriched wearable-based intervention was not more effective than the basic wearable-based intervention in its current form. Further research is needed to identify which individuals would particularly benefit from either intervention variation and explore if further personalization could increase their overall effectiveness.

### Strengths and Limitations

This research contributes to the literature by assessing the effectiveness of wearable-based interventions for reducing stress and improving well-being in a real-world context. By utilizing consumer-available wearables and investigating the impact of the interventions over several weeks in a real-life setting, where the participants are dealing with true life demands, the current findings can be generalized toward occupational settings, especially highly demanding work settings. Additionally, this study used a nested design that optimized the comparability between the control and intervention periods (ie, for analysis 1). Due to the clustered recruitment strategy and geographical distribution across teams, the potential for spillover effects between the 2 investigated wearable-based interventions was considered to be low (ie, for analysis 2).

A limitation of the study that could have caused a potential underestimation of the true effect sizes via ceiling effects [64] is the inclusion of a relatively healthy population. To at least partially account for this limitation, an LMM approach that was optimized for repeated measures (and therefore accounted for potential baseline differences between participants) was used in favor of using change scores [65]. Another limitation was that the Wearable+ intervention simulated the application of enriched feedback by providing custom-made feedback reports. It is possible that more integrated and continuously available feedback could be more user-friendly and potentially more effective.

### Comparison With Prior Work

The results of analysis 1, which showed that offering a wearable-based intervention improved stress- and well-being–related outcomes, are in line with those of prior studies that investigated the effectiveness of wearables in real-life settings. For instance, Ponzo et al [31] reported positive effects on anxiety, depression, and general well-being following a 4-week intervention that used a wearable device (BioBeam) that measured sleep, physical activity, and heart rate, as well as an app that utilized EMA and relaxation exercises based on a randomized controlled trial in a student population. Another randomized controlled trial, by Smith et al [32], showed favorable effects from a 4-week intervention on stress and anxiety, but used a wearable (Spire Stone) that primarily focuses on optimizing breathing patterns. Other studies on the effectiveness of wearable-based interventions were often performed in laboratory settings, often in young (student) populations [34]. The results of this study therefore expand upon this prior work by exploring the effectiveness of wearable-based interventions in a working population with high job demands and assessing actual changes in lifestyle outcomes and potential precursors of those in a real-life context.

We are not aware of similar prior work comparing the effectiveness of enriched and basic wearable-based interventions as in analysis 2. The difference between our enriched and basic wearable-based intervention can be broken down into the addition of (1) EMA questionnaires, (2) complementary and personalized feedback, and (3) peer support groups. The potential of combining wearable data with EMA questionnaires and complementary feedback is an emerging research topic in this field [66-68], but information about the benefits of EMA and expanded feedback next to wearable data is scarce. Similarly, there are indications that peer support groups can have a positive effect on the recovery of patients with mental health conditions [47-49] and may benefit self-care in patients with medically unexplained symptoms [46], but the effects in a preventive occupational setting are unknown. The lack of prior research on these specific topics highlights the relevance of this study, though based on our results, no clear benefits of these 3 extensions to the basic wearable-based intervention were identified.

### Conclusions

Given the evidence obtained from our analyses, offering a 5-week wearable-based intervention to police officers has small but consistent positive effects on their stress, well-being, and related MCA and self-efficacy. The results also showed that there are large interindividual differences in the outcomes that were analyzed, which may be a sign that there is potential for further assessment of possible differences in effectiveness in subgroups, as well as for improvements in the overall effectiveness via more personalized approaches. Complementing the basic “off-the-shelf” wearable-based intervention with additional EMA questionnaires, weekly personalized feedback reports, and peer support groups did not appear to improve the effectiveness of the intervention in its current form. Future research is needed to better understand how the properties of the enriched condition can be improved to increase the intervention’s effectiveness and which participants may or may not benefit from such extended feedback and support.

## Supplementary material

10.2196/60708 Multimedia Appendix 1 The original Dutch versions and English translations of the items of the Metacognitive Awareness about Sleep, Stress, and Activity Assessment questionnaire. The items fall into 3 categories that are in line with the concept of metacognitive awareness, namely awareness about responses, causes, and regulation. All 32 items are scored on a 7-point Likert scale, ranging from “completely disagree” to “completely agree.”

10.2196/60708 Multimedia Appendix 2 All other scales that were used besides the Metacognitive Awareness about Sleep, Stress, and Activity Assessment (Multimedia Appendix 1), the Mental Health Continuum Short-Form [56], and the 10-item version of Perceived Stress Scale [57] are described. The basis of and rationale for the use of these scales can be found in the Methods section.

10.2196/60708 Multimedia Appendix 3 Results of the stepwise performed linear mixed models for analysis 1, where first the confounder “wearable ownership” is included, then the main effects of the variables of interest “time” (repeated measures before and after each period) and “period” (control or intervention), and finally the interaction of time × period, the key aspect for the current analyses.

10.2196/60708 Multimedia Appendix 4 Results of the stepwise performed linear mixed models for analysis 2, where first the confounder “wearable ownership” is included, then the main effects of the variables of interest “time” (repeated measures before and after each period), “period” (control or intervention) and “group” (Wearable or Wearable+), and finally the interaction of time × period × group, which is the key aspect for these analyses.

## Abbreviations

c*R*^2^: conditional *R*^2^
EMA: ecological momentary assessment
HRV: heart rate variability
LMM: linear mixed model
MASSAA: Metacognitive Awareness about Sleep, Stress, and Activity Assessment
MCA: metacognitive awareness
MHC-SF: Mental Health Continuum-Short Form
m*R*^2^: marginal *R*^2^
PSS-10: Perceived Stress Scale, 10-item version
