# Supplementary material for: The Effects of Self-Monitoring Using a Smartwatch and Smartphone App on Stress Awareness, Self-Efficacy, and Well-Being–Related Outcomes in Police Officers: Longitudinal Mixed Design Study
Source: JMIR Mhealth Uhealth. 2025 Jan 28;13:e60708. doi: 10.2196/60708 (PMC11793834; doi:10.2196/60708)
Supplement: Multimedia Appendix 1 [file mhealth-v13-e60708-s001.docx]

# Multimedia Appendix 1

*This appendix contains the original Dutch versions and English translations of the items of the Metacognitive Awareness about Sleep, Stress and Activity Assessment (MASSAA) questionnaire. The items fall into three categories that are in line with the concept of metacognitive awareness, namely awareness about responses, causes and regulation. All 32 items are scored on a 7-point Likert scale, ranging from “completely disagree” to “completely agree”.*

## Stress Awareness & Cause

| Dutch version | English version |
| --- | --- |
| Ik herken het bij mezelf als ik veel stress heb | I recognize it in myself when I have too much stress |
| Ik herken het bij mezelf als ik langere tijd veel stress heb | I recognize it in myself when I have too much stress for a longer period of time |
| Ik herken bij mezelf signalen van overbelasting | I recognize signs of overload in myself |
| Ik merk het aan mezelf als ik na mijn dienst (meer) hersteltijd nodig heb | I notice in myself when I need (more) recovery time after my shift |
| Ik merk het aan mezelf als ik tijdens mijn dienst (meer) hersteltijd nodig heb | I notice in myself when I need (more) recovery time during my shift |
| Ik weet welke omstandigheden of situaties ik stressvol vind | I know which circumstances I find stressful |
| Ik weet wat bij mij zorgt voor een stressvolle periode | I know what causes a stressful period for me |
| Als ik gestrest ben weet ik waar dat door komt | When I am stressed I know what caused it |
| Als ik na een dienst (meer) moet herstellen weet ik waardoor dat komt | When I need (more) time to recover after a shift I know what caused it |

## Stress Regulation

| Dutch version | English version |
| --- | --- |
| Ik weet wat ik zelf (het beste) kan doen om de stress te verminderen bij een heel stressvol moment | I know what I can do (best) to reduce stress during a very stressful moment |
| Ik weet wat ik zelf (het beste) kan doen om de stress te verminderen als ik langere tijd stress heb | I know what I can do (best) to reduce stress when I experience stress for a longer period of time |
| Ik weet wat ik zelf (het beste) kan doen om te herstellen van een heel stressvol moment | I know what I can do (best) to recover during from a very stressful moment |
| Ik weet wat ik zelf (het beste) kan doen om te herstellen van langdurige stress | I know what I can do (best) to recover when I experience stress for a longer period of time |

## Sleep Awareness & Cause

| Dutch version | English version |
| --- | --- |
| Ik weet hoe mijn lichaam reageert wanneer ik slecht/niet voldoende heb geslapen | I know how my body reacts when I have slept badly/not enough |
| Ik herken bij mezelf als ik slecht/niet voldoende heb geslapen | I recognize it in myself when I have slept badly/not enough |
| Ik weet welk effect slaapgebrek heeft op mijn functioneren | I know what effect sleep deprivation has on my functioning |
| Ik weet welk effect slaapgebrek heeft op hoe ik mij voel | I know what effect sleep deprivation has on how I feel |
| Ik weet welke stressvolle omstandigheden of situaties ervoor kunnen zorgen dat ik slecht/niet voldoende slaap | I know what stressful circumstances or situations can cause me to sleep poorly/not enough |
| Ik weet welk effect stressvolle omstandigheden of situaties hebben op mijn slaap | I know what effect stressful circumstances or situations have on my sleep |
| Ik weet welk effect slecht/niet voldoende slapen heeft op hoe ik omga met stressvolle omstandigheden of situaties | I know what effect poor/not getting enough sleep has on how I deal with stressful circumstances or situations |

## Sleep Regulation

| Dutch version | English version |
| --- | --- |
| Ik weet wat ik zelf (het beste) kan doen om beter te slapen | I know what I can do (best) to sleep better |
| Ik weet wat voor mij werkt om beter te kunnen slapen | I know what works for me to sleep better |
| Ik weet wat ik kan doen om te ontspannen zodat ik goed in slaap kan komen | I know what I can do to relax so I can get a good night's sleep |

## Activity Awareness & Cause

| Dutch version | English version |
| --- | --- |
| Ik weet hoe mijn lichaam reageert wanneer ik niet voldoende beweeg | I know how my body reacts when I am not physically active enough |
| Ik herken bij mezelf als ik niet voldoende heb bewogen | I recognize it in myself when I have not been physically active enough |
| Ik weet welk effect voldoende bewegen heeft op mijn functioneren | I know what effect sufficient physical activity has on my functioning |
| Ik weet welk effect voldoende bewegen heeft op hoe ik mij voel | I know what effect sufficient physical activity has on how I feel |
| Ik weet welke stressvolle omstandigheden of situaties ervoor kunnen zorgen dat ik niet voldoende beweeg | I know what stressful circumstances or situations can prevent me from not being physically active enough |
| Ik weet welk effect stressvolle omstandigheden of situaties hebben op mijn beweeggedrag | I know what effect stressful circumstances or situations have on my physical activity behaviour |
| Ik weet welk effect voldoende bewegen heeft op hoe ik omga met stressvolle omstandigheden of situaties | I know what effect getting enough physical activity has on how I deal with stressful circumstances or situations |

## Activity Regulation

| Dutch version | English version |
| --- | --- |
| Ik weet wat ik zelf (het beste) kan doen om meer te bewegen | I know what I can do (best) to get more physical activity |
| Ik weet wat voor mij werkt om meer te bewegen | I know what works for me to be more physically active |
