# Supplementary material for: The Effects of Self-Monitoring Using a Smartwatch and Smartphone App on Stress Awareness, Self-Efficacy, and Well-Being–Related Outcomes in Police Officers: Longitudinal Mixed Design Study
Source: JMIR Mhealth Uhealth. 2025 Jan 28;13:e60708. doi: 10.2196/60708 (PMC11793834; doi:10.2196/60708)
Supplement: Multimedia Appendix 2 [file mhealth-v13-e60708-s002.docx]

# Multimedia Appendix 2

*In this appendix, all other scales that were used besides the MASSAA (Appendix 1), the Mental Health Continuum Short-Form (MHC-SF)[56] and 10-item version of Perceived Stress Scale (PSS-10)[57] are described. The basis of and rationale for the use of these scales can be found in the methods section of the full paper.*

## Self-efficacy behavior change

*These items are scored on a 5-point Likert scale, with answer options “completely disagree”, “disagree”, “neutral”, “agree”, “completely agree”.*

| Dutch version | English version |
| --- | --- |
| In hoeverre ben je het eens met de volgende stellingen? | To what extent do you agree with the following statements? |
| Ik heb er vertrouwen in dat ik er zelf voor kan zorgen dat ik voldoende slaap | I am confident that I can make sure I get enough sleep myself |
| Ik heb er vertrouwen in dat ik er zelf voor kan zorgen dat ik genoeg beweeg | I am confident that I can make sure I am enough physically active myself |
| Ik heb er vertrouwen in dat ik er zelf voor kan zorgen dat ik voldoende herstel na mijn dienst | I am confident in my own ability to ensure that I recover sufficiently after my shift |
| Ik heb er vertrouwen in dat ik er zelf voor kan zorgen dat ik voldoende herstel tijdens mijn dienst | I am confident in my own ability to ensure that I recover sufficiently during my shift |

## Self-efficacy stress resilience

*These items are scored on a 5-point Likert scale, with answer options “completely disagree”, “disagree”, “neutral”, “agree”, “completely agree”.*

| Dutch version | English version |
| --- | --- |
| In hoeverre ben je het eens met de volgende stellingen? | To what extent do you agree with the following statements? |
| Ik herstel snel na tegenslag | I recover quickly after setbacks |
| Ik vind het makkelijk om mijn leven weer op te pakken als er iets vervelends gebeurt | I find it easy to resume my life when something unpleasant happens |
| Ik heb niet veel tijd nodig om te herstellen van stressvolle situaties | I don't take much time to recover from stressful situations |

## Self-efficacy coping

*These items are scored on a 5-point Likert scale, with answer options “completely disagree”, “disagree”, “neutral”, “agree”, “completely agree”.*

| Dutch version | English version |
| --- | --- |
| In hoeverre ben je het eens met de volgende stellingen? | To what extent do you agree with the following statements? |
| Ik kan me goed concentreren onder druk | I can concentrate well under pressure |
| Ik kan goed omgaan met vervelende gevoelens | I can deal well with unpleasant feelings |
| Zelfs als ik onder grote druk sta, blijf ik kalm | Even when under great pressure, I remain calm |

## Self-efficacy task

*These items are scored on a 5-point Likert scale, with answer options “completely disagree”, “disagree”, “neutral”, “agree”, “completely agree”.*

| Dutch version | English version |
| --- | --- |
| In hoeverre ben je het eens met de volgende stellingen? | To what extent do you agree with the following statements? |
| Ik ben fysiek sterk genoeg om mijn taken naar behoren uit te voeren | I am physically strong enough to perform my duties properly |
| Ik heb vertrouwen in mijn eigen vaardigheden als politiemedewerker | I am confident in my own abilities as a police officer |
| Ik heb voldoende kennis om mijn werk te doen | I have sufficient knowledge to do my job |
| Ik ben mentaal sterk genoeg om mijn taken naar behoren uit te voeren | I am mentally strong enough to perform my duties properly |

## Recovery after work

*These items are scored on a 5-point Likert scale, with answer options “completely disagree”, “disagree”, “neutral”, “agree”, “completely agree”.*

| Dutch version | English version |
| --- | --- |
| Geef aan in hoeverre je het eens of oneens bent met de onderstaande stellingen. Beantwoord de stellingen voor de afgelopen maand. Na mijn werk ... | Please indicate the extent to which you agree or disagree with the statements below. Answer the statements for the past month. After my work ... |
| ... vergeet ik mijn werk | ... I forget my work |
| ... denk ik helemaal niet meer aan mijn werk | ... I don't think about my work at all |
| ... neem ik afstand van mijn werk | ... I take distance from my work |
| ... kan ik de eisen van mijn werk loslaten | ... I can let go of the demands of my work |

## Recovery during work

*These items are scored on a 5-point Likert scale, with answer options “completely disagree”, “disagree”, “neutral”, “agree”, “completely agree”.*

| Dutch version | English version |
| --- | --- |
| De onderstaande stellingen gaan over herstel tijdens het werk in de afgelopen maand. Geef aan in hoeverre je het eens of oneens bent met de onderstaande stellingen. | The statements below are about recovery while working in the past month. Please indicate the extent to which you agree or disagree with the statements below. |
| Tijdens pauzes kan ik goed herstellen | During breaks I can recover well |
| Na een pauze ben ik weer vol energie | After a break I am full of energy again |
| Na een pauze heb ik weer zin om aan het werk te gaan | After a break, I feel like getting back to work again |

## Sleep issues

*These items are scored on a 5-point Likert scale, with answer options “never”, “almost never”, “sometimes”, “often”, “always”.*

| Dutch version | English version |
| --- | --- |
| De afgelopen maand … | Over the past month ... |
| ... had ik last van te vroeg wakker worden | ... I suffered from waking up too early |
| ... had ik moeite met doorslapen | ... I had trouble sleeping through |
| ... had ik moeite met inslapen | ... I had trouble falling asleep |
