# Supplementary material for: The Effects of Self-Monitoring Using a Smartwatch and Smartphone App on Stress Awareness, Self-Efficacy, and Well-Being–Related Outcomes in Police Officers: Longitudinal Mixed Design Study
Source: JMIR Mhealth Uhealth. 2025 Jan 28;13:e60708. doi: 10.2196/60708 (PMC11793834; doi:10.2196/60708)
Supplement: Multimedia Appendix 3 [file mhealth-v13-e60708-s003.docx]

# Multimedia Appendix 3

*Results of the stepwise performed Linear Mixed Models (LMM) for analysis 1, where first the confounder ‘wearable ownership’ is included, then the main effects of the variables of interest ‘time’ (repeated measures before and after each period) and ‘period’ (control or intervention), and finally the interaction of time*period, which is the key aspect for the current analyses.*

### Metacognitive Awareness (MCA) of Stress – Awareness & Cause

Table 1. The results for MCA Stress Awareness & Cause.

| Independent variable | MCA Stress Awareness & Cause | | | | | |  |
| --- | --- | --- | --- | --- | --- | --- | --- |
|  | Step 1^a^ | | Step 2^b^ | | Step 3^c^ | | |
|  | β | p | β | p | β | p | |
|  |  |  |  |  |  |  | |
| Intercept | -0.07 | .55 | -0.20 | .13 | -0.13 | .36 | |
| Ownership | 0.18 | .34 | 0.18 | .34 | 0.18 | .34 | |
| Time |  |  | 0.13 | .09 | -0.02 | .83 | |
| Period |  |  | 0.13 | .09 | -0.02 | .83 | |
| Time*Period |  |  |  |  | 0.30 | .04^d^ | |

^a^Marginal R^2^ .01, conditional R^2^ .58

^b^Marginal R^2^ .02, Δ marginal R^2^ .01, conditional R^2^ .59, Δ conditional R^2^ .01

^c^Marginal R^2^ .02, Δ marginal R^2^ .02, conditional R^2^ .59, Δ conditional R^2^ .00, Hedge’s g 0.30 (0.01 to 0.58)

^d^p-value is statistically significant at p<.05 level

### Metacognitive Awareness (MCA) of Stress – Regulation

Table 2. The results for MCA Stress Regulation.

| Independent variable | MCA Stress Regulation | | | | | |  |
| --- | --- | --- | --- | --- | --- | --- | --- |
|  | Step 1^a^ | | Step 2^b^ | | Step 3^c^ | | |
|  | β | p | β | p | β | p | |
|  |  |  |  |  |  |  | |
| Intercept | -0.13 | .31 | -0.34 | .01^d^ | -0.24 | .08 | |
| Ownership | 0.32 | .11 | 0.32 | .11 | 0.32 | .11 | |
| Time |  |  | 0.22 | .00^e^ | 0.01 | .90 | |
| Period |  |  | 0.22 | .00^e^ | 0.01 | .90 | |
| Time*Period |  |  |  |  | 0.40 | -.00^f^ | |

^a^Marginal R^2^ .02, conditional R^2^ .66

^b^Marginal R^2^ .05, Δ marginal R^2^ .03, conditional R^2^ .69, Δ conditional R^2^ .03

^c^Marginal R^2^ .06, Δ marginal R^2^ .01, conditional R^2^ .70, Δ conditional R^2^ .01, Hedge’s g 0.41 (0.16 to 0.66)

^d^p-value is statistically significant at p<.05 level

^e^p-value is statistically significant at p<.001 level

^f^p-value is statistically significant at p<.01 level

### Metacognitive Awareness (MCA) of Sleep – Awareness & Cause

Table 3. The results for MCA Sleep Awareness & Cause.

| Independent variable | MCA Sleep Awareness & Cause | | | | | |  |
| --- | --- | --- | --- | --- | --- | --- | --- |
|  | Step 1^a^ | | Step 2^b^ | | Step 3^c^ | | |
|  | β | p | β | p | β | p | |
|  |  |  |  |  |  |  | |
| Intercept | -0.16 | .20 | -0.29 | .03^d^ | -0.25 | .07 | |
| Ownership | 0.38 | .05^d^ | 0.38 | .05^d^ | 0.38 | .05^d^ | |
| Time |  |  | 0.13 | .06 | 0.05 | .63 | |
| Period |  |  | 0.13 | .06 | 0.05 | .63 | |
| Time*Period |  |  |  |  | 0.17 | .24 | |

^a^Marginal R^2^ .03, conditional R^2^ .60

^b^Marginal R^2^ .04, Δ marginal R^2^ .01, conditional R^2^ .60, Δ conditional R^2^ .00

^c^Marginal R^2^ .05, Δ marginal R^2^ .01, conditional R^2^ .61, Δ conditional R^2^ .01, Hedge’s g 0.17 (-0.11 to 0.45)

^d^p-value is statistically significant at p<.05 level

### Metacognitive Awareness (MCA) of Sleep – Regulation

Table 4. The results for MCA Sleep Regulation.

| Independent variable | MCA Sleep Regulation | | | | | |  |
| --- | --- | --- | --- | --- | --- | --- | --- |
|  | Step 1^a^ | | Step 2^b^ | | Step 3^c^ | | |
|  | β | p | β | p | β | p | |
|  |  |  |  |  |  |  | |
| Intercept | -0.08 | .55 | -0.27 | .04^d^ | -0.25 | .08 | |
| Ownership | 0.19 | .35 | 0.19 | .35 | 0.19 | .35 | |
| Time |  |  | 0.20 | .00^e^ | 0.14 | .10 | |
| Period |  |  | 0.20 | .00^e^ | 0.14 | .10 | |
| Time*Period |  |  |  |  | 0.11 | .38 | |

^a^Marginal R^2^ .01, conditional R^2^ .68

^b^Marginal R^2^ .03, Δ marginal R^2^ .02, conditional R^2^ .71, Δ conditional R^2^ .03

^c^Marginal R^2^ .03, Δ marginal R^2^ .00, conditional R^2^ .71, Δ conditional R^2^ .00, Hedge’s g 0.11 (-0.13 to 0.35)

^d^p-value is statistically significant at p<.05 level

^e^p-value is statistically significant at p<.01 level

### Metacognitive Awareness (MCA) of Physical Activity – Awareness & Cause

Table 5. The results for MCA Sleep Awareness & Cause.

| Independent variable | MCA Sleep Awareness & Cause | | | | | |  |
| --- | --- | --- | --- | --- | --- | --- | --- |
|  | Step 1^a^ | | Step 2^b^ | | Step 3^c^ | | |
|  | β | p | β | p | β | p | |
|  |  |  |  |  |  |  | |
| Intercept | -0.22 | .08 | -0.38 | .00^d^ | -0.31 | .02^d^ | |
| Ownership | 0.53 | .01^d^ | 0.53 | .01^d^ | 0.53 | .01^e^ | |
| Time |  |  | 0.16 | .01^e^ | 0.02 | .84 | |
| Period |  |  | 0.16 | .01^e^ | 0.02 | .84 | |
| Time*Period |  |  |  |  | 0.29 | .03^d^ | |

^a^Marginal R^2^ .07, conditional R^2^ .64

^b^Marginal R^2^ .08, Δ marginal R^2^ .01, conditional R^2^ .66, Δ conditional R^2^ .02

^c^Marginal R^2^ .09, Δ marginal R^2^ .01, conditional R^2^ .67, Δ conditional R^2^ .01, Hedge’s g 0.30 (0.04 to 0.57)

^d^p-value is statistically significant at p<.01 level

^e^p-value is statistically significant at p<.05 level

### Metacognitive Awareness (MCA) of Sleep – Regulation

Table 6. The results for MCA Sleep Regulation.

| Independent variable | MCA Sleep Regulation | | | | | |  |
| --- | --- | --- | --- | --- | --- | --- | --- |
|  | Step 1^a^ | | Step 2^b^ | | Step 3^c^ | | |
|  | β | p | β | p | β | p | |
|  |  |  |  |  |  |  | |
| Intercept | -0.20 | .10 | -0.39 | .00^d^ | -0.35 | .01^d^ | |
| Ownership | 0.50 | .01^d^ | 0.50 | .01^d^ | 0.50 | .01^d^ | |
| Time |  |  | 0.19 | .00^d^ | 0.11 | .26 | |
| Period |  |  | 0.19 | .00^d^ | 0.11 | .26 | |
| Time*Period |  |  |  |  | 0.17 | .21 | |

^a^Marginal R^2^ .06, conditional R^2^ .63

^b^Marginal R^2^ .08, Δ marginal R^2^ .02, conditional R^2^ .65, Δ conditional R^2^ .02

^c^Marginal R^2^ .08, Δ marginal R^2^ .00, conditional R^2^ .66, Δ conditional R^2^ .01, Hedge’s g 0.17 (-0.10 to 0.44)

^d^p-value is statistically significant at p<.01 level

### Self-Efficacy (SE) – Behavior Change

Table 7. The results for SE Behavior Change.

| Independent variable | SE Behavior Change | | | | | |  |
| --- | --- | --- | --- | --- | --- | --- | --- |
|  | Step 1^a^ | | Step 2^b^ | | Step 3^c^ | | |
|  | β | p | β | p | β | p | |
|  |  |  |  |  |  |  | |
| Intercept | -0.20 | .11 | -0.29 | .03^d^ | -0.20 | .14 | |
| Ownership | 0.50 | .01^d^ | 0.50 | .01^d^ | 0.50 | .01^d^ | |
| Time |  |  | 0.09 | .12 | -0.09 | .29 | |
| Period |  |  | 0.09 | .12 | -0.09 | .29 | |
| Time*Period |  |  |  |  | 0.36 | .00^e^ | |

^a^Marginal R^2^ .06, conditional R^2^ .73

^b^Marginal R^2^ .06, Δ marginal R^2^ .00, conditional R^2^ .73, Δ conditional R^2^ .00

^c^Marginal R^2^ .07, Δ marginal R^2^ .01, conditional R^2^ .74, Δ conditional R^2^ .01, Hedge’s g 0.37 (0.13 to 0.60)

^d^p-value is statistically significant at p<.05 level

^e^p-value is statistically significant at p<.01 level

### Self-Efficacy (SE) – Stress Resilience

Table 8. The results for SE Stress Resilience.

| Independent variable | SE Stress Resilience | | | | | |  |
| --- | --- | --- | --- | --- | --- | --- | --- |
|  | Step 1^a^ | | Step 2^b^ | | Step 3^c^ | | |
|  | β | p | β | p | β | p | |
|  |  |  |  |  |  |  | |
| Intercept | -0.10 | .43 | -0.27 | .05 | -0.20 | .15 | |
| Ownership | 0.25 | .22 | 0.25 | .22 | 0.25 | .22 | |
| Time |  |  | 0.16 | .01^d^ | 0.04 | .66 | |
| Period |  |  | 0.16 | .01^d^ | 0.04 | .66 | |
| Time*Period |  |  |  |  | 0.25 | .04^e^ | |

^a^Marginal R^2^ .01, conditional R^2^ .69

^b^Marginal R^2^ .03, Δ marginal R^2^ .02, conditional R^2^ .70, Δ conditional R^2^ .03

^c^Marginal R^2^ .03, Δ marginal R^2^ .00, conditional R^2^ .71, Δ conditional R^2^ .01, Hedge’s g 0.25 (0.01 to 0.50)

^d^p-value is statistically significant at p<.01 level

^e^p-value is statistically significant at p<.05 level

### Self-Efficacy (SE) – Coping

Table 9. The results for SE Coping.

| Independent variable | SE Behavior Change | | | | | |  |
| --- | --- | --- | --- | --- | --- | --- | --- |
|  | Step 1^a^ | | Step 2^b^ | | Step 3^c^ | | |
|  | β | p | β | p | β | p | |
|  |  |  |  |  |  |  | |
| Intercept | -0.06 | .64 | -0.12 | .38 | -0.09 | .53 | |
| Ownership | 0.15 | .46 | 0.15 | .46 | 0.15 | .46 | |
| Time |  |  | 0.06 | .33 | -0.01 | .94 | |
| Period |  |  | 0.06 | .33 | -0.01 | .94 | |
| Time*Period |  |  |  |  | 0.13 | .28 | |

^a^Marginal R^2^ .01, conditional R^2^ .72

^b^Marginal R^2^ .01, Δ marginal R^2^ .00, conditional R^2^ .72, Δ conditional R^2^ .00

^c^Marginal R^2^ .01, Δ marginal R^2^ .00, conditional R^2^ .72, Δ conditional R^2^ .00, Hedge’s g 0.13 (-0.10 to 0.37)

### Self-Efficacy (SE) – Task

Table 10. The results for SE Task.

| Independent variable | SE Task | | | | | |  |
| --- | --- | --- | --- | --- | --- | --- | --- |
|  | Step 1^a^ | | Step 2^b^ | | Step 3^c^ | | |
|  | β | p | β | p | β | p | |
|  |  |  |  |  |  |  | |
| Intercept | -0.01 | .93 | -0.18 | .20 | -0.10 | .46 | |
| Ownership | 0.03 | .89 | 0.03 | .89 | 0.03 | .89 | |
| Time |  |  | 0.16 | .01^d^ | 0.02 | .82 | |
| Period |  |  | 0.16 | .01^d^ | 0.02 | .82 | |
| Time*Period |  |  |  |  | 0.29 | .03^d^ | |

^a^Marginal R^2^ .00, conditional R^2^ .66

^b^Marginal R^2^ .01, Δ marginal R^2^ .01, conditional R^2^ .68, Δ conditional R^2^ .02

^c^Marginal R^2^ .02, Δ marginal R^2^ .01, conditional R^2^ .68, Δ conditional R^2^ .00, Hedge’s g 0.29 (0.04 to 0.54)

^d^p-value is statistically significant at p<.05 level

### Mental Health Continuum (MHC)

Table 11. The results for MHC.

| Independent variable | MHC | | | | | |  |
| --- | --- | --- | --- | --- | --- | --- | --- |
|  | Step 1^a^ | | Step 2^b^ | | Step 3^c^ | | |
|  | β | p | β | p | β | p | |
|  |  |  |  |  |  |  | |
| Intercept | 0.01 | .95 | -0.09 | .49 | 0.01 | .97 | |
| Ownership | -0.02 | .92 | -0.02 | .92 | -0.02 | .92 | |
| Time |  |  | 0.10 | .09 | -0.10 | .24 | |
| Period |  |  | 0.10 | .09 | -0.10 | .24 | |
| Time*Period |  |  |  |  | 0.40 | .00^d^ | |

^a^Marginal R^2^ .00, conditional R^2^ .72

^b^Marginal R^2^ .01, Δ marginal R^2^ .01, conditional R^2^ .73, Δ conditional R^2^ .01

^c^Marginal R^2^ .02, Δ marginal R^2^ .02, conditional R^2^ .74, Δ conditional R^2^ .01, Hedge’s g 0.40 (0.17 to 0.63)

^d^p-value is statistically significant at p<.001 level

### Recovery After Work

Table 12. The results for Recovery After Work.

| Independent variable | Recovery After Work | | | | | |  |
| --- | --- | --- | --- | --- | --- | --- | --- |
|  | Step 1^a^ | | Step 2^b^ | | Step 3^c^ | | |
|  | β | p | β | p | β | p | |
|  |  |  |  |  |  |  | |
| Intercept | -0.06 | .69 | -0.12 | .39 | -0.13 | .37 | |
| Ownership | 0.14 | .53 | 0.14 | .53 | 0.14 | .53 | |
| Time |  |  | 0.07 | .17 | 0.08 | .24 | |
| Period |  |  | 0.07 | .17 | 0.08 | .24 | |
| Time*Period |  |  |  |  | -0.03 | .77 | |

^a^Marginal R^2^ .00, conditional R^2^ .83

^b^Marginal R^2^ .01, Δ marginal R^2^ .01, conditional R^2^ .83, Δ conditional R^2^ .00

^c^Marginal R^2^ .01, Δ marginal R^2^ .00, conditional R^2^ .83, Δ conditional R^2^ .00, Hedge’s g -0.03 (-0.21 to 0.16)

^d^p-value is statistically significant at p<.05 level

### Recovery During Work

Table 13. The results for Recovery During Work.

| Independent variable | Recovery During Work | | | | | |  |
| --- | --- | --- | --- | --- | --- | --- | --- |
|  | Step 1^a^ | | Step 2^b^ | | Step 3^c^ | | |
|  | β | p | β | p | β | p | |
|  |  |  |  |  |  |  | |
| Intercept | -0.12 | .38 | -0.14 | .32 | -0.19 | .19 | |
| Ownership | 0.29 | .17 | 0.29 | .17 | 0.29 | .17 | |
| Time |  |  | 0.02 | .69 | 0.12 | .12 | |
| Period |  |  | 0.02 | .69 | 0.12 | .12 | |
| Time*Period |  |  |  |  | -0.19 | .07 | |

^a^Marginal R^2^ .02, conditional R^2^ .78

^b^Marginal R^2^ .02, Δ marginal R^2^ .00, conditional R^2^ .78, Δ conditional R^2^ .00

^c^Marginal R^2^ .02, Δ marginal R^2^ .00, conditional R^2^ .748, Δ conditional R^2^ .00, Hedge’s g -0.19 (-0.40 to 0.02)

### Sleep Issues

Table 14. The results for Sleep Issues.

| Independent variable | Sleep Issues | | | | | |  |
| --- | --- | --- | --- | --- | --- | --- | --- |
|  | Step 1^a^ | | Step 2^b^ | | Step 3^c^ | | |
|  | β | p | β | p | β | p | |
|  |  |  |  |  |  |  | |
| Intercept | 0.10 | .45 | 0.14 | .30 | 0.11 | .43 | |
| Ownership | -0.25 | .23 | -0.25 | .23 | -0.25 | .23 | |
| Time |  |  | -0.04 | .44 | 0.02 | .76 | |
| Period |  |  | -0.04 | .44 | 0.02 | .76 | |
| Time*Period |  |  |  |  | -0.13 | .23 | |

^a^Marginal R^2^ .01, conditional R^2^ .76

^b^Marginal R^2^ .02, Δ marginal R^2^ .01, conditional R^2^ .76, Δ conditional R^2^ .00

^c^Marginal R^2^ .02, Δ marginal R^2^ .00, conditional R^2^ .76, Δ conditional R^2^ .00, Hedge’s g -0.13 (-0.35 to 0.08)

### Perceived Stress Scale (PSS)

Table 15. The results for PSS.

| Independent variable | PSS | | | | | |  |
| --- | --- | --- | --- | --- | --- | --- | --- |
|  | Step 1^a^ | | Step 2^b^ | | Step 3^c^ | | |
|  | β | p | β | p | β | p | |
|  |  |  |  |  |  |  | |
| Intercept | 0.03 | .84 | 0.17 | .21 | 0.06 | .67 | |
| Ownership | -0.06 | .76 | -0.06 | .76 | -0.06 | .76 | |
| Time |  |  | -0.15 | .02^d^ | 0.09 | .33 | |
| Period |  |  | -0.15 | .02^d^ | 0.09 | .33 | |
| Time*Period |  |  |  |  | -0.46 | .00^e^ | |

^a^Marginal R^2^ .00, conditional R^2^ .68

^b^Marginal R^2^ .01, Δ marginal R^2^ .01, conditional R^2^ .69, Δ conditional R^2^ .01

^c^Marginal R^2^ .02, Δ marginal R^2^ .01, conditional R^2^ .71, Δ conditional R^2^ .02, Hedge’s g -0.46 (-0.70 to -0.22)

^d^p-value is statistically significant at p<.05 level

^e^p-value is statistically significant at p<.001 level
